# Advanced Unresectable Differentiated Thyroid Cancer With Anaplastic Transformation: A Case Report and Review of the Literature

**DOI:** 10.7759/cureus.73956

**Published:** 2024-11-18

**Authors:** Jehad Alzahrani, Suhaib Radi, Abdullah Aljabri, Mohammad Alandejani

**Affiliations:** 1 Department of Internal Medicine, Division of Endocrinology, Ministry of the National Guard-Health Affairs, Jeddah, SAU; 2 College of Medicine, King Saud Bin Abdulaziz University for Health Sciences, King Abdullah International Medical Research Center, Jeddah, SAU

**Keywords:** adtc, anaplastic transformation, lenvatinib, neoadjuvant, unresectable

## Abstract

Papillary thyroid carcinoma (PTC) is the most common subtype of thyroid cancer (TC). Although surgery and radioactive iodine therapy (RAI) generally yield favorable outcomes, advanced cases with extensive local invasion and metastases pose significant challenges. We report the case of a 65-year-old male with advanced, inoperable PTC characterized by extensive local invasion and distant metastases. A whole-body radioiodine scan revealed no iodine-avid disease. However, following recombinant human thyrotropin (rhTSH) preparation, the patient developed severe compressive symptoms, necessitating glucocorticoid treatment. Lenvatinib monotherapy was initiated, resulting in initial symptom relief. Unfortunately, the disease rapidly progressed due to anaplastic transformation of the PTC, ultimately leading to the patient's death. This report highlights the challenges of managing advanced differentiated TC (DTC) and the risk of aggressive transformation into anaplastic TC (ATC), even with targeted therapy. It underscores the critical need for close monitoring and a multidisciplinary approach. Further research is needed to explore the role of targeted therapies, such as lenvatinib, in altering tumor biology and their long-term effects on disease progression.

## Introduction

Thyroid cancer (TC) is the most prevalent endocrine malignancy [1] and the ninth most common cancer globally, with a particularly high incidence among women, second only to breast cancer [2,3]. In Saudi Arabia, the incidence and prevalence of TC have steadily increased, with rates of 10.1% and 12.9%, respectively [4,5]. Papillary thyroid carcinoma (PTC) is the most frequent subtype of TC [5]. Although women have a higher incidence, men exhibit higher mortality rates [4,6]. Differentiated thyroid carcinoma (DTC), which includes PTC, generally has an excellent prognosis, with a five-year survival rate of 98.3% [7]. Based on many guidelines, the standard treatment involves surgery, followed by radioactive iodine (RAI) therapy, depending on the risk of recurrence. Cases refractory to RAI may be treated with systemic chemotherapy or targeted therapies, such as tyrosine kinase inhibitors (TKIs) like sorafenib and lenvatinib [8-10]. However, approximately 13-15% of DTC cases develop into advanced DTC (ADTC), characterized by local invasion, bulky cervical nodes, or distant metastases. These cases are more challenging to manage and have a poor prognosis [11,12]. We discuss a case of ADTC complicated by extensive local invasion and distant metastasis, highlighting the complexities in management and treatment outcomes.

Informed consent was obtained from the patient's next of kin for the publication of this report.

## Case presentation

A 65-year-old male with a history of hypertension was referred to our endocrinology clinic in Jeddah, Saudi Arabia, for the evaluation of a progressively enlarging neck mass that had been present for approximately six years. Initially, the patient had not sought medical attention, but the increasing size of the mass had prompted him to consult his primary physician. At that time, a thyroid ultrasonography had revealed multiple bilateral thyroid nodules and enlarged lymph nodes. Fine-needle aspiration (FNA) of the lymph node had confirmed papillary thyroid cancer (PTC), leading to a referral to our endocrinology service for further evaluation. On physical examination, the patient’s vital signs were stable. A neck examination revealed a right thyroid nodule measuring 3 cm and several enlarged, indurated, and fixed lymph nodes, with the largest measuring 4 cm.

**Figure 1:**
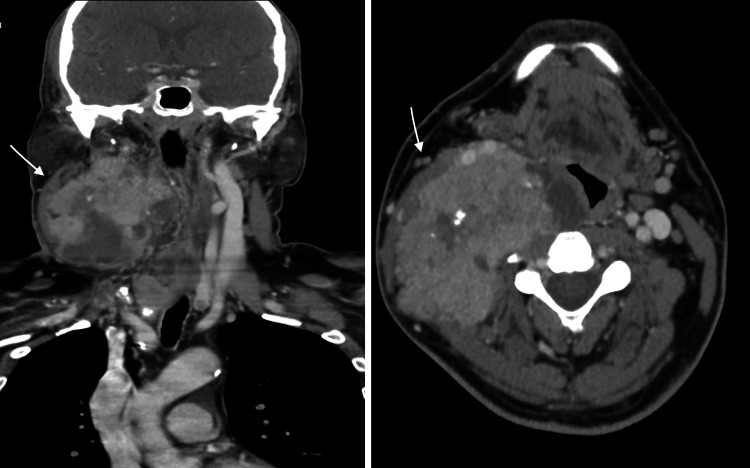
Neck CT axial and coronal views at initial evaluation The images show both thyroid lesions and the lymph node mass with significant displacement of adjacent structures, including the larynx, hypopharynx, and right carotid sheath CT: computed tomography

Laboratory test results showed normal thyroid function with normal thyroid-stimulating hormone (TSH) and free thyroxine levels. However, the thyroglobulin (Tg) level was considerably elevated, while the thyroglobulin antibody level was normal. The results of all other parameters of a comprehensive metabolic panel were within acceptable limits (Table 1). Ultrasonography of the thyroid revealed multiple bilateral nodules. The largest nodule in the right lobe measured 2.4 × 2.3 × 2.2 cm, appeared as a hypoechoic multilobulated solid nodule with central calcification, and was coded as Ti-RAD 5. An enlarged lymph node, heterogeneous with internal calcifications, measuring 6.7 × 6.3 × 4.8 cm, was also identified. FNA of the lymph node confirmed PTC. The immunohistochemical markers, including PAX8, galectin, thyroid-transcription factor 1 (TTF-1), HBME-1, cyclin D1, and Tg were positive. Neck CT revealed tumor encasement of the right common carotid artery, carotid bifurcation, and proximal external and internal carotid arteries. The right internal jugular vein was also encased and not visualized in the upper portion, suggesting total occlusion or tumor invasion (Figure 1). Chest CT revealed bilateral lung nodules, consistent with metastatic disease.

**Table 1:** Initial laboratory workup FT4: free thyroxine; Tg: thyroglobulin; TgAb: thyroglobulin antibody; TSH: normal thyroid-stimulating hormone

Parameter	Result	Normal range
TSH	2.26 IU/L	0.4–4.5 IU/L
FT4	11.85 pmol/L	9–19 pmol/L
Tg	>2500 ng/mL	0.2–70 ng/mL
TgAb	5.29 IU/mL	5–100 IU/mL

**Figure 2:**
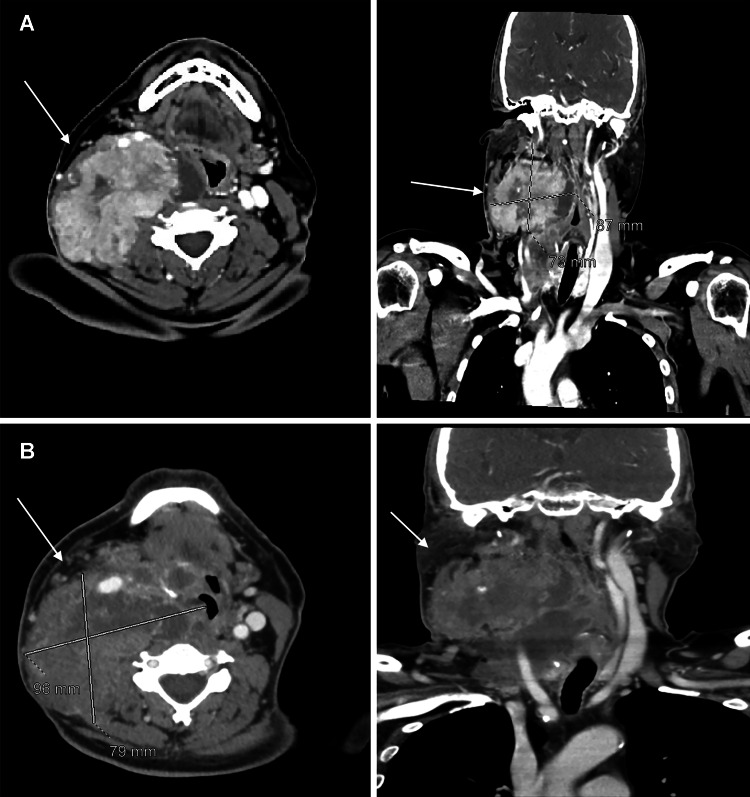
A) Neck CT with axial and coronal views prior to the evaluation with WBS and rhTSH. B) Neck CT with axial and coronal views one and a half months after using rhTSH, demonstrating an increase in the size of both lesions, primarily in the thyroid CT: computed tomography; rhTSH: recombinant human thyroid stimulating hormone; WBS: whole-body scan

Given the advanced nature of the PTC, including extensive lymph node metastases, the recommended treatment was total thyroidectomy with central and right-sided neck lymph node dissection. However, based on the neck CT findings, the patient was deemed unsuitable for surgery. He sought a second medical opinion, which confirmed the diagnosis of inoperable advanced PTC. He returned to our hospital three months later with a worsening neck mass, measuring approximately 5.3 × 5.4 × 6.2 cm. The right lymph node measured about 9.2 × 8.2 × 7.2 cm (Figure 2A). A multidisciplinary tumor board recommended a diagnostic whole-body scan (WBS) using the thyrotropin alfa [recombinant human thyroid stimulating hormone (rhTSH)] protocol to evaluate radioiodine avidity. If the disease was radioiodine-avid, neoadjuvant radioiodine ablation therapy would be considered to reduce the tumor size. Following the whole-body scan, the patient experienced severe headache and compressive symptoms, including shortness of breath and changes in voice, as well as a feeling of mass enlargement. This was presumed to be attributable to the thyroid inflammatory response to thyrotropin alfa, which necessitated oral dexamethasone treatment to alleviate all symptoms. Unfortunately, no imaging was performed during these symptoms as the patient refused an emergency room visit and the symptoms resolved considerably with dexamethasone. However, about a month and a half later, a CT of the neck showed a progressive increase in size to 7.9 × 7.5 from 5.7 × 5.4 cm, with the size of the right lymph node remaining almost unchanged (Figure 2B).

Nevertheless, the WBS indicated non-avid disease. Based on this result, and after a thorough discussion with the patient, lenvatinib therapy was initiated, with external beam radiation as a potential option if the response to lenvatinib was inadequate. The patient began treatment with lenvatinib at a dose of 14 mg daily.

**Figure 3:**
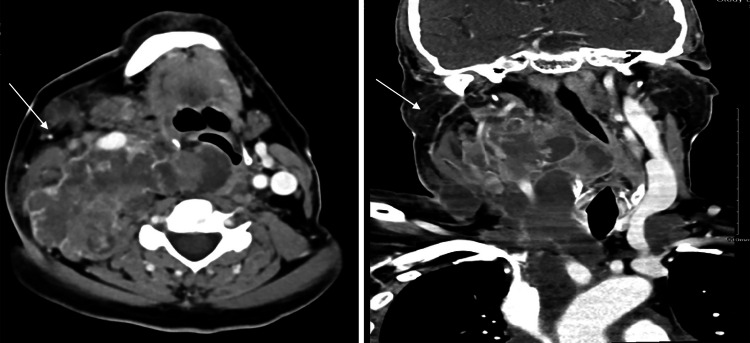
Neck CT axial and coronal views during the emergency room visit following the deterioration of symptoms on lenvatinib treatment and anaplastic transformation The images show further progression in size and mass effect with airway deviation CT: computed tomography

At the three-week follow-up, the patient reported a subjective reduction in the size of the neck mass and no adverse effects from lenvatinib. However, a follow-up CT scan one month later showed a significant progression of the thyroid lesion and cervical lymphadenopathy, which now appeared more necrotic. At the eight-week follow-up, the patient demonstrated marked clinical improvement, with a decrease in compressive symptoms. His thyroglobulin levels decreased from >2500 ng/mL to 1390 ng/mL. Unfortunately, five months after starting lenvatinib therapy, the patient was hospitalized with stridor and shortness of breath. His TSH levels were markedly elevated at 39.7 IU/L and dexamethasone and levothyroxine were administered to manage these symptoms. The patient’s white blood cell (WBC) count increased, likely due to intravenous administration of dexamethasone and sepsis. Subsequent imaging revealed further progression of the thyroid lesions, now measuring 9.4 × 8.8 cm (previously 7.9 × 7.5 cm), with a newly identified enlarged lymph node on the right, causing mass effect and deviation of the airway (Figure 3).

A tracheostomy was performed, and a biopsy of the thyroid revealed anaplastic thyroid carcinoma (ATC). Histopathological analysis of the biopsy sample revealed positive staining for thyroid transcription factor 1 and negative staining for thyroglobulin. The patient was then admitted to the ICU, where he developed complications, including pulmonary embolism and hospital-acquired pneumonia, which ultimately led to his demise.

## Discussion

Neoadjuvant therapy for unresectable ADTC - a review

TC management typically involves surgery; however, some patients may present with inoperable disease because of patient- or disease-related factors. There is no clear definition for ADTC in the literature or American Thyroid Association (ATA) guidelines. Russell et al. define ADTC as involving "local invasion, bulky cervical nodes, distant metastases, or recurrent disease", in their review of surgical approaches to advanced thyroid cancer [12]. Unfortunately, most guidelines do not provide specific recommendations for neoadjuvant therapy in these cases, while a few could be inferred from either a metastatic disease or an advanced unresectable recurrent disease. Our review identified radiotherapy and chemotherapy as the primary neoadjuvant options for ADTC.

Two retrospective, non-randomized studies from Slovenia were featured in an article review that examined systemic chemotherapy as a neoadjuvant treatment for 45 patients with advanced DTC; these studies demonstrated that systemic chemotherapy (vinblastine, vinblastine with doxorubicin, or other regimens) achieved tumor size reductions greater than 50% in 44.8% and 40% of cases, respectively. Surgical resection rates R0/R1 (complete resection/microscopic remnant) were 86.2% (R0 = 51.7%) and 75% (R0 = 12.5%), respectively [13], suggesting that neoadjuvant systemic chemotherapy may be effective for treating ADTC. Furthermore, more recent trials, such as the SELECT trial, highlight the growing use of targeted chemotherapies such as TKIs, particularly lenvatinib, for cases resistant to RAI [14]. A systematic review revealed partial remission in 84.3% of ADTC cases treated with TKIs, with R0/R1 resections achieved in 78.1% of cases [15]. These findings indicate the significant efficacy of TKIs, particularly lenvatinib, as a neoadjuvant option for unresectable ADTC.

Additionally, external beam radiotherapy (EBRT) has been investigated as an initial treatment for unresectable ADTC in a retrospective cohort of 33 patients. During follow-up, 51.1% of patients showed a partial response, which made them eligible for surgery after a median period of 13.3 months. Post-surgical follow-up demonstrated favorable outcomes in both overall survival and progression-free survival. However, radiation toxicity is a crucial factor in the management of locally advanced diseases. Severe dysphagia occurred in 36% of patients, requiring percutaneous endoscopic gastrostomy, while 12.1% experienced tracheal invasion complications, leading to upper airway obstruction and the necessity for a tracheostomy. Additionally, radiation-induced locoregional changes combined with the advanced nature of the disease increased the risk of more severe surgical complications [16]. Unfortunately, the role of RAI as a neoadjuvant treatment has not been thoroughly evaluated.

Overall, we recommend the use of lenvatinib to treat unresectable ADTC, either alone or in combination with EBRT, followed by salvage surgery. However, further research is needed to confirm this approach.

The use and safety of rhTSH in ADTC

The ATA recommends using rhTSH for RAI preparation in patients with low or intermediate risk of DTC. This recommendation is based on its demonstrated non-inferiority to thyroid hormone withdrawal (THW) and its benefits for short-term quality of life. However, the ATA does not provide guidelines for using rhTSH in ADTC, except in cases where THW is contraindicated [8]. In our case, the patient experienced a substantial increase in tumor size following the administration of rhTSH, which improved with dexamethasone treatment. We attributed this response to the effects of thyrotropin. A similar sudden enlargement of DTC was reported by an observational study involving rhTSH in ADTC, where three of 21 patients experienced acute dyspnea and choking following rhTSH administration. The symptoms were alleviated with hydrocortisone, and notably, none of the patients experienced similar symptoms after THW [17].

A review of several case reports and series on rhTSH use in metastatic DTC also identified rapid tumor enlargement that responded to glucocorticoid treatment, indicating that the swelling may be due to inflammation-related edema rather than tumor growth [18]. Similar local symptoms have been documented, including headaches and neurological issues in patients with brain and spinal metastases, dyspnea in those with lung metastases, and bone pain in those with bone metastases [17,18]. Given these safety concerns, the ATA recommends conducting brain and spine MRIs before initiating rhTSH therapy if metastases are present. If metastases are detected, high-dose glucocorticoid treatment should be started before rhTSH administration and continued for up to 72 h thereafter [8]. Despite these adverse effects, previous studies indicate that rhTSH is effective in treating both ADTC and metastatic DTC [17,18]. A recent meta-analysis further supports its efficacy, demonstrating comparable outcomes to THW in metastatic thyroid cancer treatment [19]. Based on these findings, rhTSH can be used in ADTC, provided that a full assessment of patient risk is conducted. Patients with tumors in confined anatomical spaces should receive glucocorticoids before rhTSH to reduce the risk of acute swelling. However, randomized controlled trials are still needed to directly compare the efficacy and safety of rhTSH versus THW in ADTC.

ADTC and ATC

ATC was identified in the final core tissue biopsy, which differed from the initial FNA and lymph node biopsy results showing PTC. This discrepancy could be explained by one of the following three possibilities: misdiagnosis or missed ATC in the initial FNA, the transformation of PTC to ATC, or the coexistence of PTC and ATC. A retrospective study of 18 patients with ATC examined their initial FNAs with subsequent histopathology; seven showed PTC background histology following surgical resection, three showed a PTC cytological component, and one with initial FNA showed PTC [20]. Vinette et al. suggested that in cases of early focal ATC transformation, if the FNA only captures the PTC component, inadequate sampling may lead to false-positive PTC diagnoses. A sudden rapid change in DTC behavior should prompt further evaluation [21].

ATC can arise from DTC in either the thyroid or metastatic areas [22-25], and overexpression of p53 may play a role in this transformation [26]. PAX8 is another marker found in up to 60% of ATC cases, and ATA guidelines recommend using both P53 and PAX8 expression in immunohistochemical analysis to support the diagnosis. However, the presence of thyroid-lineage markers such as TTF-1 and TG typically indicates DTC and is expected to be absent in ATC [22].

Given the potential for transformation, the coexistence of both DTC and ATC is a recognized phenomenon, reported in up to 5% of cases [27-29]. We believe that, in our case, coexisting active tumors arose after the transformation of ADTC. This is supported by the clinical presentation of sudden rapid tumor growth following six years of indolence, elevated TG levels, the initial response to lenvatinib, and immunohistochemical findings. Specifically, TTF-1 positivity supports DTC, while the presence of both P53 and PAX8 suggests possible dedifferentiation to ATC.

Lenvtinib’s role in the transformation of DTC into ATC

Lenvatinib has an established role in treating RAI-resistant DTC and ADTC, with favorable outcomes [8]. Although it can also be used for ATC, the response rate is notably lower; Iwasaki et al. reported a response rate of only 17.4% [30]. We observed that during the initial course of treatment, lenvatinib primarily targeted DTC, leading to early tumor shrinkage and symptom improvement. However, owing to its poor efficacy against ATC, the disease progressed rapidly. A similar observation was made in a previous similar case report. Nonetheless, another possible explanation proposed by the author is that the function of TKIs in signal blocking caused a mutation, and as a result, lenvatinib caused an ATC transformation [31]. A case series described three patients who received lenvatinib for 5.1 months, 13.1 months, and 5.09 years respectively. All of them experienced a sudden deterioration in their clinical condition, characterized by elevated WBC count, Glasgow Coma Scale-Flexion, and interleukin-6 level, which was suspected to be linked to ATC transformation. Leukocytosis was identified as a potential marker for ATC transformation, especially when coupled with a worsening clinical status. The authors recommended increased vigilance and further investigation in such cases [32]. Although our patient's WBC count was elevated, it is difficult to interpret this as a marker of ATC transformation as the patient was also receiving intravenous dexamethasone and may have had sepsis.

In summary, although lenvatinib has demonstrated efficacy in managing DTC, there is scarce data on its long-term effects and role in treating ATC. Given the potential for rapid ATC progression, close monitoring is essential. If a patient’s condition worsens, additional diagnostic investigations, including repeat FNA, should be promptly conducted to assess for ATC transformation. Further research is needed to gain deeper insights into the long-term safety and efficacy of lenvatinib in treating patients with coexisting DTC and ATC.

## Conclusions

We presented a complex case of ADTC, which is particularly challenging when the disease becomes unresectable and continues to progress despite targeted therapies. Given the lack of a standard definition for ADTC and the absence of clear treatment protocols, a multidisciplinary, personalized approach is essential for optimizing outcomes, especially considering the potential for aggressive transformation into ATC, as observed in this case. Based on our review, the use of lenvatinib as a targeted therapy can provide significant improvement. However, while it offers a viable therapeutic option for managing advanced cases, its role in altering tumor biology, particularly the risk of transformation to ATC, warrants further investigation. Moreover, incorporating EBRT and considering salvage surgery in eligible patients may further enhance treatment outcomes, particularly for patients with localized disease. Overall, the management of ADTC requires careful monitoring and a comprehensive, multidisciplinary treatment approach. We strongly recommend further research into targeted therapies like lenvatinib to optimize care in this patient population.
